# LINE-1 Hypomethylation During Primary Colon Cancer Progression

**DOI:** 10.1371/journal.pone.0018884

**Published:** 2011-04-14

**Authors:** Eiji Sunami, Michiel de Maat, Anna Vu, Roderick R. Turner, Dave S. B. Hoon

**Affiliations:** 1 Department of Molecular Oncology, John Wayne Cancer Institute, Santa Monica, California, United States of America; 2 Department of Surgical Pathology, Saint John's Health Center, Santa Monica, California, United States of America; University of Hong Kong, Hong Kong

## Abstract

**Background:**

Methylation levels of genomic repeats such as long interspersed nucleotide elements (LINE-1) are representative of global methylation status and play an important role in maintenance of genomic stability. The objective of the study was to assess LINE-1 methylation status in colorectal cancer (CRC) in relation to adenomatous and malignant progression, tissue heterogeneity, and TNM-stage.

**Methodology/Principal Findings:**

DNA was collected by laser-capture microdissection (LCM) from normal, adenoma, and cancer tissue from 25 patients with TisN0M0 and from 92 primary CRC patients of various TNM-stages. The paraffin-embedded tissue sections were treated by *in-situ* DNA sodium bisulfite modification (SBM). LINE-1 hypomethylation index (LHI) was measured by absolute quantitative analysis of methylated alleles (AQAMA) realtime PCR; a greater index indicated enhanced hypomethylation. LHI in normal, cancer mesenchymal, adenoma, and CRC tissue was 0.38 (SD 0.07), 0.37 (SD 0.09), 0.49 (SD 0.10) and 0.53 (SD 0.08), respectively. LHI was significantly greater in adenoma tissue compared to its contiguous normal epithelium (*P* = 0.0003) and cancer mesenchymal tissue (*P*<0.0001). LHI did not differ significantly between adenoma and early cancer tissue of Tis stage (*P* = 0.20). LHI elevated with higher T-stage (*P*<0.04), was significantly greater in node-positive than node-negative CRC patients (*P* = 0.03), and was significantly greater in stage IV than all other disease stages (*P*<0.05).

**Conclusion/Significance:**

By using *in-situ* SBM and LCM cell selection we demonstrated early onset of LINE-1 demethylation during adenomatous change of colorectal epithelial cells and demonstrated that LINE-1 demethylation progression is linear in relation to TNM-stage progression.

## Introduction

Approximately 17–18% of the human genome consists of long interspersed nucleotide element (LINE-1) repeats. Roughly 500,000 truncated and 3,000 to 5,000 full length LINE-1 sequences are present throughout the genome [1]. In normal somatic cells, LINE-1s are heavily methylated, restricting activities of retrotransposal elements and thus preventing genomic instability [2], [3]. LINE-1 sequences are moderately CpG rich, and most methylated CpGs are located in the 5′ region and can behave as internal promoters [2]. LINE-1 methylation status is thought to represent the genome-wide DNA methylation status, since LINE-1 sequences are highly repeated, widely interspersed human retrotransposons. Various studies have shown a relation of important genomic events in colorectal carcinogenesis to LINE-1 methylation. For example, 18q loss of heterozygosity (LOH) (+) colorectal cancer (CRC) show a lower mean LINE-1 methylation level [4]. An inverse relation has been reported between LINE-1 methylation and microsatellite instability (MSI)+/CpG island methylator phenotype (CIMP)+/BRAF V600E mutation CRC [5], [6]. There has recently been reported a significant inverse correlation between levels of LINE-1 methylation and the total number of chromosomal aberrations, including both losses and gains in gastrointestinal stromal tumors (GIST) [7]. Regarding CRC, Bariol et al. revealed that the global DNA hypomethylation level was greater in neoplastic lesions (including hyperplastic polyps and adenoma) than in normal mucosa [8].

An important problem in the assessment of LINE-1 methylation status in CRC is that CRC is highly heterogeneous and contains various infiltrating cells such as stromal cells, lymphocytes, and blood vessels. The stromal component and extent of lymphocyte infiltration varies greatly among CRCs. This heterogeneity may confound molecular analysis, especially of LINE-1 methylation status, as LINE-1 is methylated in normal cells. In order to obtain accurate interpretations, it is therefore necessary to specifically isolate tumor cells from tissue specimens. Early stage CRC primary tumor analysis without histopathology-directed microdissection for assessment of genomic methylation status is not accurate. This is particularly a problematic issue when assessing CRC progression and when comparing adenoma to cancer tissue. Robust and accurate techniques for such analyses do not exist; therefore, we developed a practical approach. We utilized laser–capture microdissection (LCM) to harvest the cells of interest directly without contamination of non-cancer cells. Sodium bisulfite modification (SBM) of genomic DNA is a commonly used method to discern methylation status of CpG islands [9]. Conventional SBM methods result into 84% to 96% loss of sample DNA [10]. This significant loss of template DNA necessitates high volumes of starting tissue sample. To reduce the loss of sample DNA from cells collected by LCM, we developed an *in-situ* SBM protocol. DNA was modified while the captured cells are still attached on the LCM cap.

Numerous studies have been reported identifying CRC-associated epigenetic aberrations such as promoter region hypermethylation of tumor suppressor genes. Another characteristic alteration in CRC regarding DNA methylation status is global hypomethylation [11], [12]. The progression of LINE-1 hypomethylation level during the development of CRC malignancy and the progression of disease has not been rigorously assessed. Determining the histopathology associated with the onset of loss of LINE-1 methylation and the trend of LINE-1 methylation along the axis of disease stage progression could further clarify the role of LINE-1 hypomethylation in CRC disease. In this study, LCM, *in-situ* SBM, and absolute quantification of methylated alleles (AQAMA) were utilized in combination on specimens from patients with adenomas containing *in situ* invasive adenocarcinoma (Tis) and patients with more advanced CRC. This allowed successful accurate analysis of LINE-1 hypomethylation levels during primary tumor progression between progressive T-stages, node positive versus node negative disease, and distant metastatic versus local and locoregional disease.

## Results

### AQAMA linearity for LINE-1 methylation level assessment

**Figure 1:**
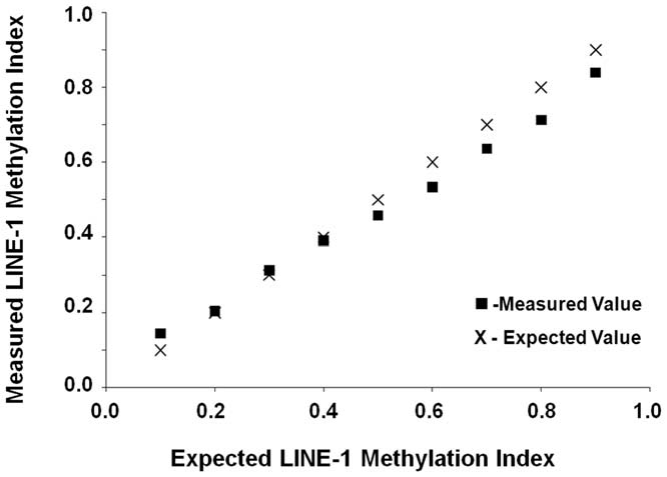
AQAMA LINE-1 assay accuracy. Plot of measured (boxes) and expected (x) values showing accuracy of the AQAMA LINE-1 assay.

First, we evaluated the accuracy of AQAMA in assessing various levels of LINE-1 methylation. The major advantage of AQAMA is that the quantitative measurement of methylated and unmethylated alleles is performed in a single PCR reaction, providing excellent control compared to two separate PCR reactions for methylated and unmethylated allele analysis. The quantification is absolute with standard curves to determine the copy number. For a negative control, the assay reaction contained universal unmethylated control DNA that was synthesized as described in a previously published study [13]. For a positive control, we used universal methylated control DNA extracted from peripheral blood lymphocytes from a healthy donor and treated with *sssI methyltransferase.* For this study, we prepared stepwise mixtures of the methylated and unmethylated cDNA standard and measured them as samples with unknown methylation level. The linearity of AQAMA assay for LINE-1 methylation level (**Figure 1**) was evaluated by Pearson’s coefficient of linearity: 0.99 (*P*<0.001), which showed high accuracy in discriminating LINE-1 methylation level differences.

### Optimization of *in-situ* SBM settings

**Figure 2:**
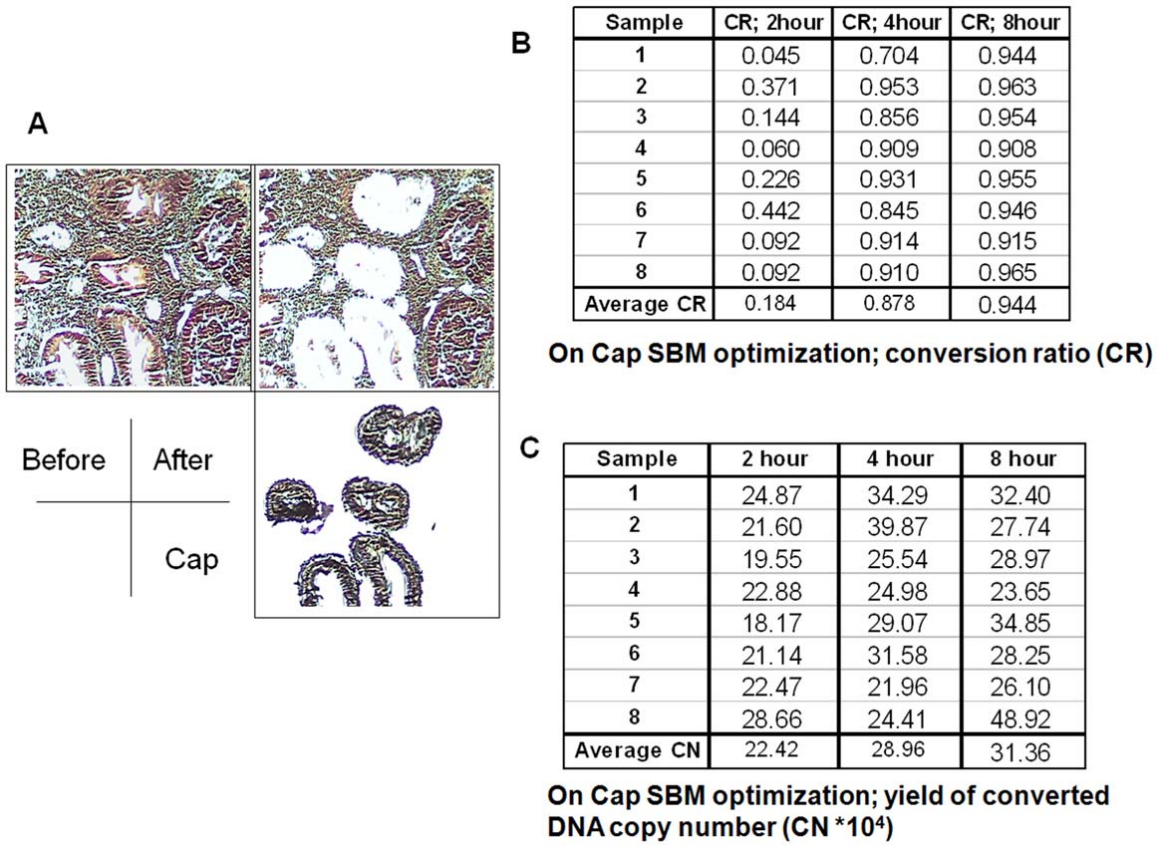
Representations of LCM combined with *in-situ* SBM optimization studies. **A** shows specific pick-up of target cells by LCM. **B** and **C** show results for DNA conversion rate (%) and for DNA content (×10^4^ copy numbers), respectively, in 8 different samples at 3 different incubation periods.

LCM was performed to harvest cells of interest (**Figure 2a**) to obtain accurate representation of cells in question for successful AQAMA measurement. Three different sample tissue areas of 10^4^, 10^5^ and 10^6^ µm^2^ harvested from 4 µm-thick paraffin-embedded archival tissue (PEAT) were tested. Through analysis, we established that 2×10^5^ µm^2^ of 4 µm-thick PEAT results in about 10^5^ copy numbers of LINE-1 DNA. This provided significant reproducible levels of DNA LINE-1 methylation assay. After LCM, *in-situ* SBM was optimized in a similar manner as was performed in the previous studies of on-slide SBM using specific genomic sequence amplification [14]. The conversion rates of modified DNA by *in-situ* SBM were 18.4±14.9%, 87.8±7.8% and 94.4±2.1% at incubation setting of 60°C for 2, 4 and 8 hrs, respectively (**Figure 2b**). The conversion rates increased with increase in duration of incubation, whereby after 8 hrs it reached about 95%. The yield of total DNA (modified and unmodified DNA) did not decrease with increasing incubation time up to 8 hrs (**Figure 2c**). From these results, an incubation setting of 60°C for 8 hrs was chosen as optimal for *in-situ* SBM.

### LINE-1 hypomethylation levels during CRC progression

**Figure 3:**
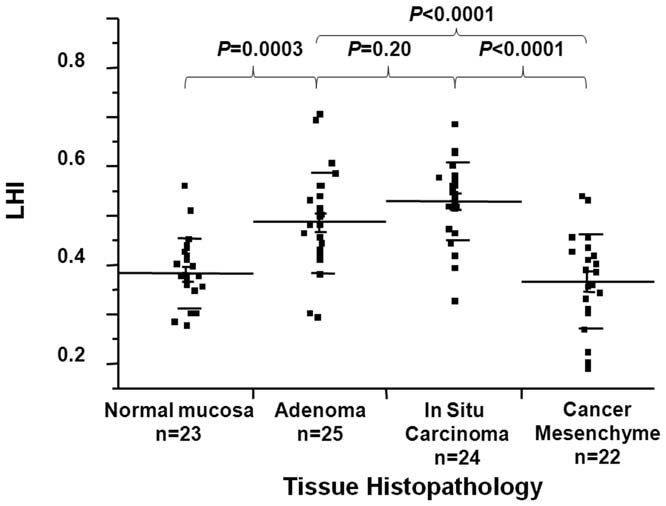
LHI assessment in normal large bowel epithelium, adenomatous cells, cancer cells (Tis) and CRC mesenchymal cells.

Twenty-five patients with TisN0M0 lesions with presence of normal, adenoma (low or intermediate grade) and Tis cancer cells on the same tissue section were selected. Using LCM, four different tissue samples (normal mucosa, adenoma, cancer, and cancer mesenchymal tissue) were collected from each patient. *In-situ* SBM and LINE-1 AQAMA assay were performed on each sample. The level of LINE-1 hypomethylation (LHI) was calculated as Q_unmeth_/(Q_unmeth_ + Q_meth_), where Q_unmeth_ and Q_meth_ are the absolute copy numbers of unmethylated and methylated LINE-1, respectively. In normal mucosa adjacent to tumor lesions and cancer mesenchyme, the average LHI was 0.382 and 0.366, respectively. There was no difference of LHI between cancer mesenchyme and normal mucosa adjacent to tumor. LHI was significantly greater in the contiguous adenoma and cancer tissue of early stage than in normal mucosa (mean LHI = 0.49, 0.52, and 0.38, respectively) (**Figure 3**). From these results, we concluded that LINE-1 hypomethylation occurs in the early stage of CRC tumorigenesis. Furthermore, these experiments demonstrated the necessity of using detailed procedures for DNA collection when analyzing LINE-1 methylation levels, such as target cell isolation, by LCM. On the basis of this, LCM was used for DNA sample collection for all experiments in our studies.

### LINE-1 hypomethylation and tissue heterogeneity

**Figure 4:**
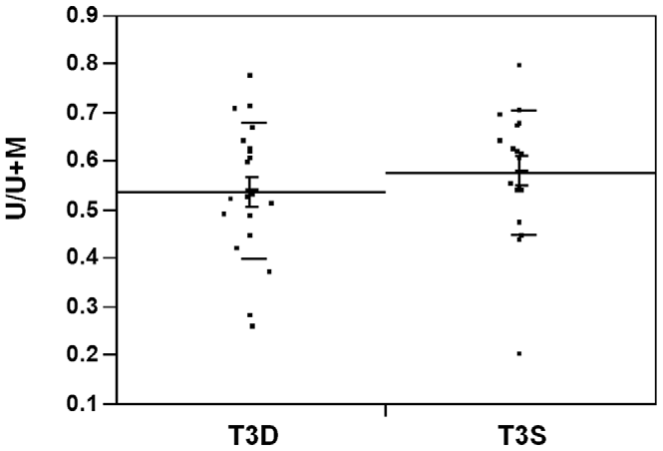
LHI analysis of CRC tumors. Measured LHI values in T3N0 CRC tumors comparing cells obtained from the surface luminal tumor area to the deep invasion tumor area.

In the analysis of LHI of normal, cancer mesenchymal, adenoma, and CRC tissue, was 0.38 (SD 0.07), 0.37 (SD 0.09), 0.49 (SD 0.10) and 0.53 (SD 0.08), respectively. To test whether there is heterogeneity of LINE-1 hypomethylation within a tumor, 20 T3N0 tumors were selected for analysis. Sample DNA was collected from the luminal surface and the deepest invasional site of each tumor. LHI of those two loci were compared (**Figure 4**). The mean LHI was 0.58 in the surface and 0.54 in the deepest loci. LHIs were similar in the surface and in the deepest loci and tumor heterogeneity of LINE-1 hypomethylation was not observed.

### LINE-1 hypomethylation and CRC stage

**Figure 5:**
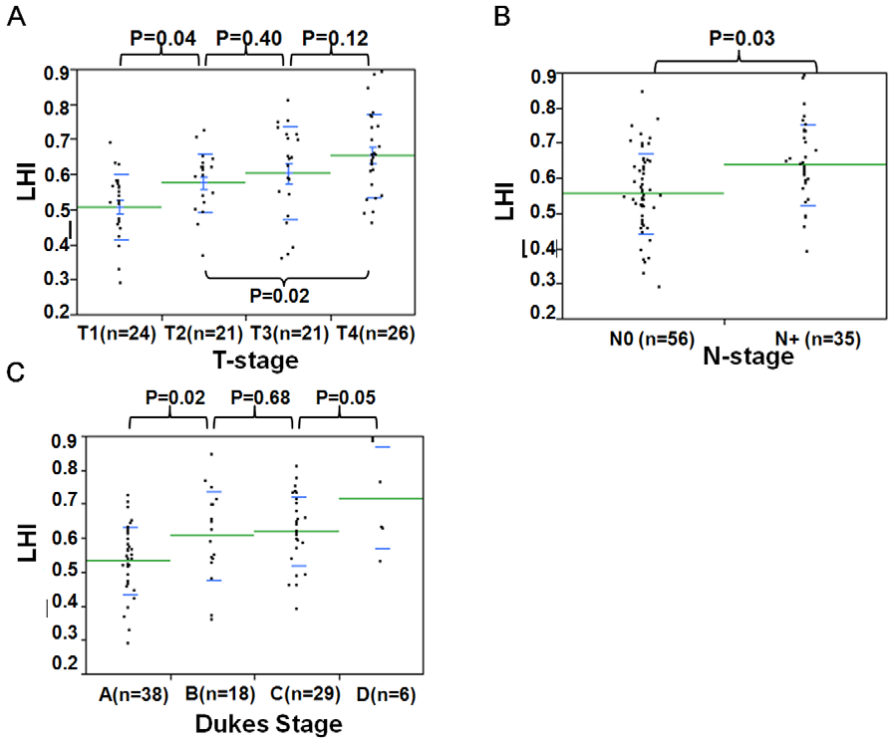
Measured LHI values comparisons between clinical disease stage classifications. In A, B and C differences are shown between Duke’s stage, T-stage and N-stage, respectively.

**Figure 6:**
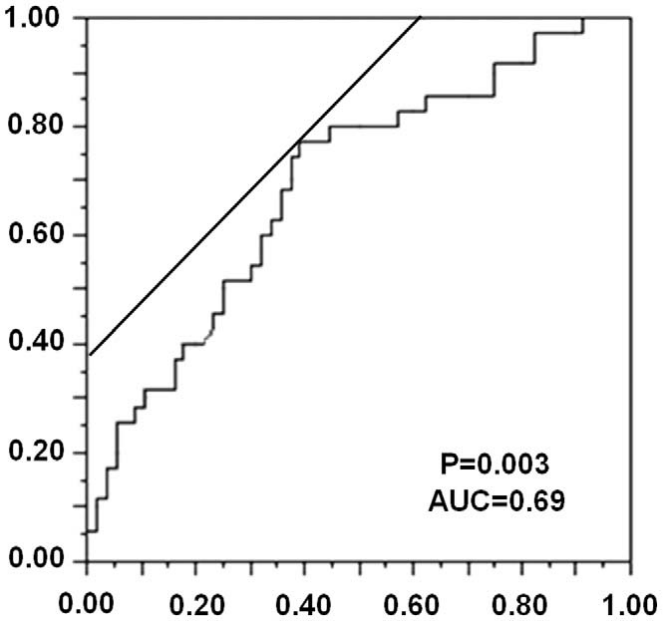
Receiver-operator characteristic (ROC) curve indicating the discriminatory accuracy of presence of nodal involvement by LHI. The x-axis represents 1-specificity and the y-axis sensitivity. The area under the curve (AUC) was calculated to 0.69. P = 0.003.

To assess the alteration of LINE-1 hypomethylation levels according to tumor progression, the correlation between Dukes stages and LHI was analyzed. Dukes A (n = 38), Dukes B (n = 18), and Dukes C (n = 29) samples were selected (**Figure 5c**). The mean LHI in Dukes A, B, and C was 0.533, 0.607, and 0.621, respectively. Dukes B and C tumors showed significantly greater LHI than Dukes A tumors. The relation between LINE-1 methylation and T-stage (T1 (n = 24), T2 (n = 21), T3 (n = 21), and T4 (n = 26)) as well as N-stage (N0 (n = 57) and N1 (n = 35)) were analyzed. The increase of LHI according to tumor invasion (**Figure 5a**) and the difference of LHIs between lymph node positive and negative cases (**Figure 5b**) were assessed. The mean LHI of T1, T2, T3, and T4 was 0.50, 0.58, 0.60, and 0.65, respectively. LHI was significantly greater (*P* = 0.03) for cases with positive lymph nodes (LHI = 0.64) than negative lymph nodes (LHI = 0.54). The prediction of nodal status would be the most clinically relevant and therefore a receiver operating curve (ROC) was analyzed to distinguish if node metastasis positive can be a prognostic indicator of the primary CRC by means of LHI (**Figure 6**). T-stage advancement correlated significantly with increasing LHI (*P* = 0.01). We also analyzed cases with distant disease spread (Dukes D, n = 6), and these cases showed significantly greater LHI compared to Dukes C CRCs (*P* = 0.05) alone and to all other Duke stages (*P*<0.0001). A ROC was plotted to show the accuracy for prediction of nodal involvement by LHI (**Figure 6**). This showed that the predictive value had a sensitivity of 0.77 with a specificity of 0.62. There was significantly lower LHI for right-sided versus left-sided colon (*P*<0.05). However, no significant differences were observed between LHI for differentiation status or gender.

These analyses showed a positive linear relation between progression of LINE-1 hypomethylation and CRC stage progression.

## Discussion

The alteration of DNA methylation patterns has been studied in many types of cancer [15]. Hypermethylation of specific cancer-associated gene promoter regions and global DNA hypomethylation during tumor progression has been now shown as a significant property of cancers [16], [17]. Hypomethylation of the LINE-1 DNA repeat which forms 20% of the genome has been suggested as a surrogate for global DNA hypomethylation [5], [18], [19], [20]. Yang et al. showed that genome-wide methylation can be estimated by assessment [21]. Alu repeats are SINEs, more complex, and diverse than LINEs. Reliable methylation assays to study Alu are not well substantiated. An accurate assay representative for global methylation phenomenon does not exist; however, LINE-1 may serve well as a surrogate.

LINE-1 hypomethylation is not specific for cancer cells, and about one third of LINE-1 is unmethylated in normal mucosa and cancer mesenchymal cells [22], [23]. The possible contamination of specimen DNA that consists of cancer along with mesenchymal cells, such as fibroblasts or lymphocytes, may therefore cause inaccurate results. For this reason, especially regarding LINE-1 methylation analysis, accurate isolation of target cells is highly important. To minimize the contamination and obtain cells of interest only, we successfully integrated LCM in LINE-1 methylation analyses in this study. We assessed *in-situ* SBM combined with LCM as an approach to improve the DNA methylation assay efficiency, which enables evaluation of cells from specific histopathologically defined target regions of very early stage primary CRC development. A recent study has provided some evidence that results are comparable whether macrodissection or LCM is used [24]. However, this is highly dependent on the sensitivity of the assay. The concept of LCM, however, is state-of-the-art and superior to macrodissection when small targeted tissue input specimen for DNA analysis is desired. First, when early-stage small lesions need to be assessed and only small areas of specific cancer cells are available for analysis, the microscopy-guided cell pick-up provides precision and control of the input sample DNA. Second, LINE-1 methylation is not cancer-specific and, as our results show, is found in cancer stromal tissue. With the current understanding of the tumor microenvironment and tumor stromal heterogeneity, it is highly important to perform LCM analysis for specific tissue DNA retrieval. Also, LINE-1 methylation would be expected to vary in adjacent “normal” cells of a primary tumor since light microscopy pathology analysis is not always accurate to identify early stage cancer cell transformation.

*In-situ* SBM was designed based on on-slide SBM [14] which was adapted from a concept as reported by Nuovo et al [25]. On-slide SBM is a powerful procedure for the methylation analysis using PEAT; however, after on-slide SBM the specimen becomes too adhesive to the glass slide, making it difficult for LCM to efficiently capture target cells at times. *In-situ* SBM eliminates the numerous DNA isolation and purification steps of classic SBM, which are the main cause of DNA loss when assessing low amounts of DNA from limited number of cells. Our study describes *in situ* treatment of tissue/cells directly by bisulfite assay integrated with LCM. A limitation of the assay is that analysis of methylation status of single cells still remains difficult due to the DNA degrading nature of the SBM protocol and limited DNA for methylation analysis.

Combining LCM, *in-situ* SBM, and AQAMA assay, LINE-1 hypomethylation was demonstrated in low to intermediate grade adenoma, an early stage of colorectal tumorigenesis as previously shown [5], [6]. A significant decrease in LINE-1 methylation was measured comparing adenoma with the adjacent normal epithelium. No significant difference was shown when the adenoma was compared to the contiguous cancerous Tis stage tissue which was also recently reported in by Kwon et al [26]. This result differs from studies by Ibrahim et al. and by Irahara et al. that found significant differences of LHI between colorectal adenomas and CRC [24], [27]. It is important to note that these studies did not specify the TNM-stage of the samples. The non-significant difference between adenoma and cancer tissues in our study is explained by the use of a heterogeneous group of cancer tissues from the earliest cancer stage (*in situ* carcinoma, Tis). Our results confirm that LINE-1 methylation levels vary in different TNM-stages. When LHI in adenomas were compared to more advanced stage CRC samples in our study the differences were significant (data not shown). This demonstrates that unmethylated LINE-1 may not play an important role during the process when adenoma cells gain their first invasive activity.

The increased heterogeneity found in larger, advanced lesions is an indication that there exist subgroups of CRC that are able to maintain methylation of LINE-1. On the other hand, we observed that two Dukes stage D cases had an LHI of 0.9 which was only seen in this subgroup. Heterogeneity is a known clinical problem as disease outcome varies widely for CRC patients with the same TNM-stage. The diversity of the LHIs within the various stages in our study may reflect this. The mechanisms that control maintenance of LINE-1 methylation and how they influence tumor progression is still unknown.

In a large study by Baba et al [28], a relation to disease stage has been described with higher LINE-1 demethylation in more advanced disease although the differences were very minor. Also, according to the study’s results, LINE methylation levels were higher in stage II disease compared to stage I. This study analyzed whole tissue sections without using microdissection. One of the reasons that differences were small and stage II showed higher LINE-1 methylation may be the degree of mesenchymal cell DNA contamination in the specimen DNA. Our results demonstrated that not only adjacent normal mucosa, but also mesenchymal cells in cancer, show about one-third of LINE-1 hypomethylation. This supports our emphasis that specific target cell isolation is necessary. Prognostic value of LINE-1 methylation levels in CRC has been reported [29]; however, variation between tumors may be a result of the stromal/mesenchymal component contamination [30].

Our study shows a clear stepwise positive linear relation of loss of LINE-1 methylation to T-, N-, as well as M-stage. The levels of LINE-1 demethylation may therefore be helpful in several clinical situations. Preoperative analysis of CRCs biopsy material that can predict T-stage (Tis vs. T1 or T1 vs. T2) may be of use to decide whether endoscopic resection (i.e. transanal mucosal excision of rectal cancers) may be attempted versus open resection surgery, which is associated with much higher morbidity/mortality rates. N-stage prediction could be used to select CRC patients to test the effectiveness of neoadjuvant therapy. Furthermore, M-stage prediction may be used as a biomarker to undertake further metastatic preoperative imaging with PET-CT scan in addition to the standard work-up. To assess whether preoperative tumor biopsy material from the luminal side is representative, we assessed tumor heterogeneity. The comparison between the deepest part and the surface of the tumor revealed that tumor heterogeneity of LINE-1 methylation status is relatively subtle. The results suggest that CRC biopsy specimens collected during colonoscopy are representative and can be used for assessment of a tumor’s LINE-1 methylation status analysis. The mean LHI differed significantly between node positive and node negative CRCs; whereby ROC analysis showed discriminatory results. There was overlap of LHI values between the N+ and N0 categories and the sensitivity and specificity were relatively low. This was also seen for T- and M-stage discrimination. Larger studies are needed to determine the utility of LHI as a single biomarker or combination with other biomarkers.

**Figure 7:**
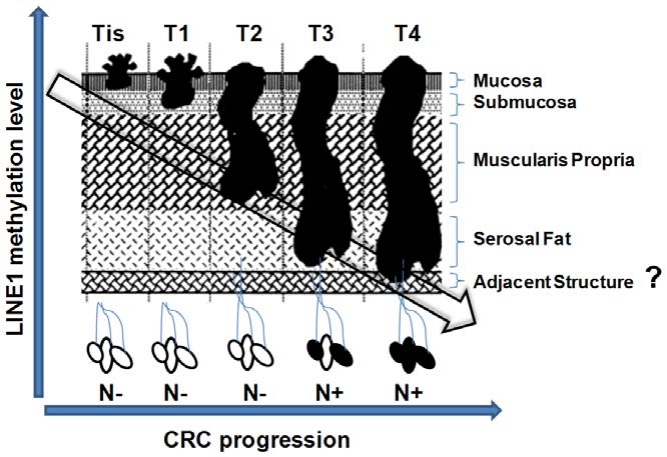
Representation of the relation between decreasing LINE-1 methylation and CRC progression.

In conclusion, the study demonstrated that LINE-1 methylation status correlates with pathological stage of CRC. Furthermore, its accurate analysis with LCM or equivalent techniques is necessary, as supportive mesenchymal tissue surrounding tumor cells clearly can influence results. The onset of LINE-1 demethylation occurs very early when normal colorectal mucosa develops into an adenoma with low or intermediate dysplasia. The results showed a clear and significant linear correlation of progression of loss of methylation LINE-1 element to progression of CRC disease (**Figure 7**) with regard to T- as well as N- and M-stage. These findings suggest continuous loss of genomic methylation during CRC development, which indicates that high epigenomic and thus genomic events are key features of tumor progression.

## Materials and Methods

### Measurement of LINE-1 hypomethylation

The methylation status of LINE-1 was evaluated by AQAMA assay for accurate assessment of CpG island methylation levels [31]. Before performing the AQAMA assay of LINE-1 methylation status, SBM was applied on each DNA sample as previously described [32]. AQAMA requires one forward and one reverse primer which will amplify the target sequence independent from the methylation status, as those forward and reverse primer sets do not contain any CpG. The methylation status is assessed by two minor-groove-binding (MGB) molecule containing probes (Applied Biosystems): one methylation specific and one unmethylation specific. The 5’ primer, 3’ primer, methylation-specific probe and unmethylation-specific probe are listed as follows: 5’-GGGTTTATTTTATTAGGGAGTGTTAGA-3’ (forward), 5’-TCACCCCTTTCTTTA ACTCAAA-3’ (reverse), FAM-5’-TGCGCGAGTCGAAGT-3’-MGB-BHQ and VIC-5’-TGTGTG AGTTGAAGTAGGG-3’-MGB-BHQ. The reaction mixture for each AQAMA PCR consisted of DNA template, 0.4 µmol/L of the forward and reverse primer, 1.4 units of *iTaq* DNA polymerase (Bio-Rad, Hercules, CA), 350 µmol/L of each deoxynucleotide triphosphate and 0.025 pmol of each MGB probe with 5 mmol/L Mg^2+^. PCR amplification was performed with pre-cycle heat activation of DNA polymerase at 95°C for 10 min, followed by 40 cycles of denaturation at 95°C for 15 sec, annealing and extension at 60°C for 60 sec. The absolute copy number in each sample was determined using a standard curve established by amplifying six aliquot duplicates of templates with known copy numbers (10^*6^ to 10^*1^ copies). We have previously described the methods for synthesis of the standard sample of known copy number in the AQAMA assay [31]. For a negative control, the assay reaction contained universal unmethylated control DNA that was synthesized as described in a previously published study [13]. For a positive control, we used universal methylated control DNA extracted from peripheral blood lymphocytes from a healthy donor and treated with *sssI methyltransferase*. Briefly, *SssI methyltransferase* treated, bisulfite modified, donor PBL DNA was amplified by PCR with the primer set to create the standard sample for fully methylated LINE-1. For construction of the standard sample for unmethylated LINE-1 we used universal unmethylated control DNA, prepared as previously described [13]. The completely methylated and unmethylated PCR product was ligated into a pCR 2.1-TOPO cloning vector (Invitrogen); the clones were transformed into *Escherichia coli* DH5-a cells; and cultures were expanded as described previously [33]. Plasmids containing the target gene were purified and quantified by UV spectrophotometry. This was used to make dilution series of known copy number to construct standard curves for absolute quantification. All quantitative PCR assays were performed in a blinded fashion without knowledge of specimen identity. Mean values were calculated from triplicate reactions. LINE-1 hypomethylation index (LHI) of each sample was calculated as follows: LINE-1 LHI  =  unmethylated copy number/(methylated copy number + unmethylated copy number).

### LCM

LCM was used for DNA sample collection for all experiments in our study. Principles and technical basis of LCM are described in detail by Fend F et al. [34]. Cells were collected with LCM system which harbours a digital camera system that detects the selected cells. This enables that the same amount of square micrometers of cells can be picked up for each sample.

### *In-situ* SBM assay optimization

To measure LINE-1 hypomethylation index after microdissection using LCM, we developed the *in-situ* SBM procedure based on previously introduced on-slide SBM technique. *In-situ* SBM was designed to analyze methylation status of genes in small amount of DNA obtained from formalin fixed PEAT. *In-situ* SBM aims to eliminate DNA isolation and purification steps that are performed before the actual SBM in the classic procedure which is the main cause of DNA loss. *In-situ* SBM contains three steps; denaturing of DNA, SBM and collection of modified DNA. First, the cells were microdissected from deparaffinized and rehydrated tissue sections that stick on the cap used in LCM are incubated in 0.2 mol/L NaOH at room temperature for 15 min. Then the samples on the cap are incubated in 3 mol/L sodium bisulfite solution with 0.5 mmol/L hydroquinone (pH 5) in the dark. Three incubation setting were tested regarding the conversion rates (the rate of modification of cytosine to uracil) and yields of modified DNA and incubation setting of 60°C for 8 hrs was chosen as optimal (see Results Section). After incubation, samples on the cap were rinsed with distilled water, soaked in 0.3 mol/L NaOH for 15 min to desulfonate the modified cytosines, and then desalted in distilled water at 60°C for 2 hrs. Finally, 50 µL lysis buffer containing 4 µg proteinase K, 2.5% Tween 20, 50 mmol/L Tris, and 1 mmol/L EDTA were added on the cap and incubated at 50°C for 24 hrs followed by heat deactivation of proteinase K at 95°C for 10 min. In each subsequent AQAMA reaction, 1–2 µL lysate was used as a template without DNA purification.

### Ethics Statement

PEAT samples of CRC were obtained from patients who underwent colectomy or proctectomy between 1995 and 1998 at Saint John's Health Center/ John Wayne Cancer Institute, Santa Monica, CA. The study was reviewed and approved by Saint John's Health Center/John Wayne Cancer Institute' Institutional Review Board (IRB) and Western IRB. Written consent was obtained from the patients.

### PEAT CRC specimens

All tissue specimens had been fixed in 10% buffered formalin for 24 hrs and paraffin-embedded. For LCM followed by *in-situ* SBM and LINE-1 methylation analysis by AQAMA, sections (4 µm) were cut with a microtome from each PEAT block. Cells were collected with the Veritas® automated LCM system (Life Technologies). One of the advantages of this system is that it enables control of the square micrometers of cells that are picked up for each sample. To assess the differences of LINE-1 methylation status between normal mucosa, adenoma, cancer and cancer mesenchymal connective tissue, 25 samples of early CRC in adenoma (TisN0M0) were selected by a pathologist (R.R.T.) specialized in CRC that contained all four of these tissue categories. To study malignant alteration, 94 surgically resected CRC specimens were procured to investigate the alteration of LINE-1 methylation status in accordance with cancer progression. LCM was performed on both study groups. The correlation between clinico-pathological stage such as T stage, nodal status and Dukes stage and LINE-1 methylation index were analyzed.

### Statistical analysis

All data are expressed as mean+/−S.D., and percentages as appropriate. Mean value of LHI between two groups such as adenoma and early cancer, node negative and node positive were compared using Student’s *t*-test.
